# Selective Oxidation of Glycerol by Highly Active Bimetallic Catalysts at Ambient Temperature under Base-Free Conditions**

**DOI:** 10.1002/anie.201101772

**Published:** 2011-08-24

**Authors:** Gemma L Brett, Qian He, Ceri Hammond, Peter J Miedziak, Nikolaos Dimitratos, Meenakshisundaram Sankar, Andrew A Herzing, Marco Conte, Jose Antonio Lopez-Sanchez, Christopher J Kiely, David W Knight, Stuart H Taylor, Graham J Hutchings

**Affiliations:** Cardiff Catalysis Institute, School of Chemistry, Cardiff UniversityMain Building, Park Place, Cardiff, CF10 3AT (UK); Center for Advanced Materials and Nanotechnology, Lehigh University5 East Packer Avenue, Bethlehem, PA 18015-3195 (USA); National Institute of Standards and Technology, Surface and Microanalysis Science Division100 Bureau Drive, Gaithersburg, MD 20899-8371 (USA)

**Keywords:** alloys, gold, heterogeneous catalysis, nanoparticles, platinum


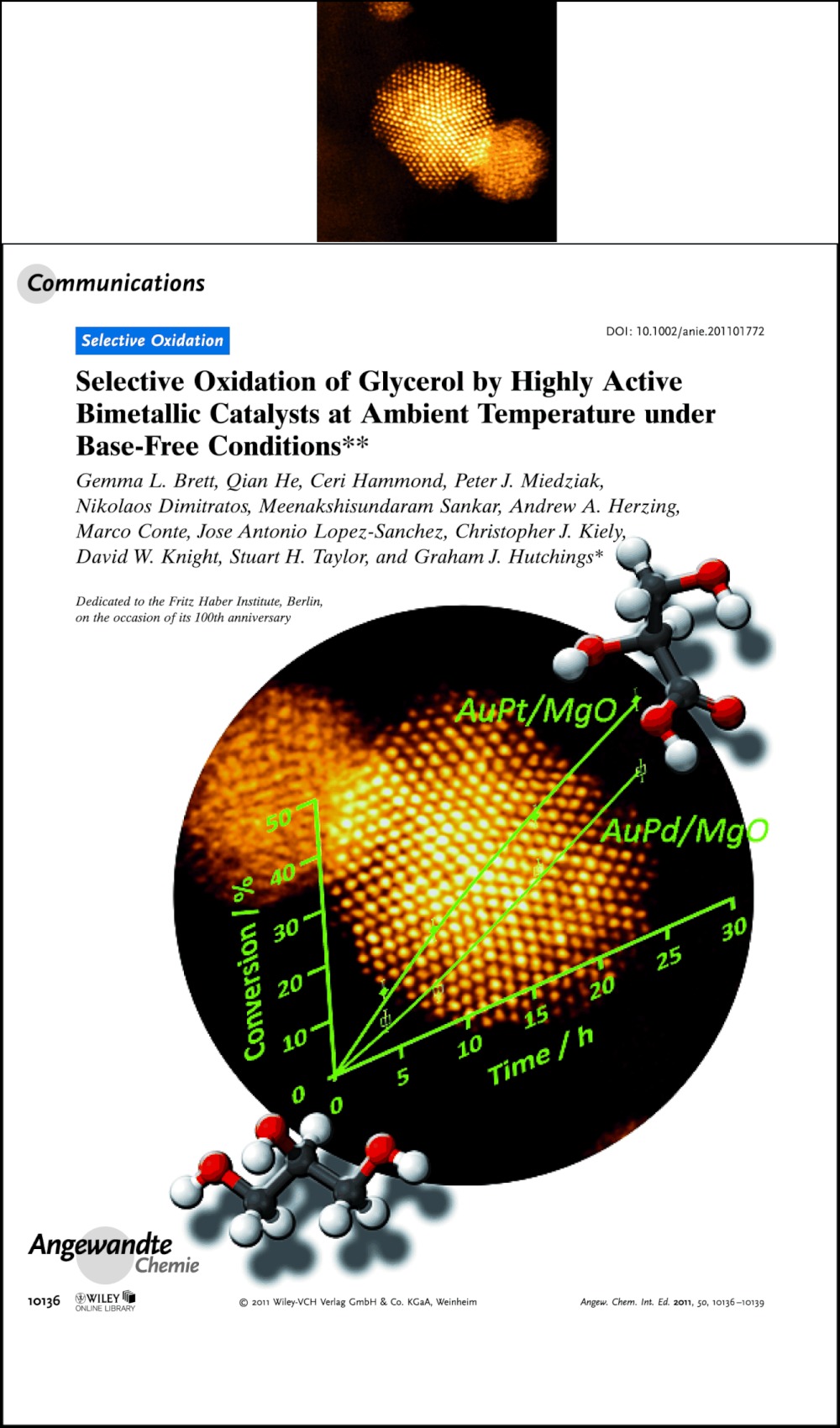


Glycerol is a by-product of the manufacture of biodiesel and its supply as a sustainable raw material is anticipated to grow steadily due to a shift towards “greener” fuels. Since glycerol provides a precursor to various industrially valuable products, such as glyceric acid, tartronic acid and hydroxyacetone, this increasing glycerol supply represents an industrially important feedstock for synthesizing such chemicals by oxidative reaction. Heterogeneous catalysts play a key role in the promotion and control of such reactions, and can also lead to “greener” synthesis routes when compared with alternatives, such as the addition of stoichiometric reagents during synthesis.

Nanoparticulate gold dispersed on a variety of oxide supports has been studied for a wide range of catalytic redox reactions,^1^–^3^ and the observation that supported gold catalysts are effective for glycerol oxidation is currently attracting considerable attention. In their initial seminal work Rossi and Prati were the first to demonstrate that supported gold nanoparticles are effective for alcohol oxidation in the presence of a base.^5^, ^6^ We subsequently extended this concept to show that glycerol could be converted into glyceric acid with a high yield using basic reaction conditions.^7^, ^8^ In contrast to palladium and platinum catalysts, gold has been shown to promote the reaction without causing over-oxidation.^8^ More recent studies of glycerol oxidation have concentrated on the role of the gold particle size,^9^ the catalyst pre-treatment,^10^ and the catalyst preparation method.^11^

While these previous studies have focused on monometallic nanoparticulate gold, we have since demonstrated that alloying gold with palladium can lead to a 25-fold enhancement in the activity of alcohol oxidation.^12^ Prati and co-workers^13^ have recently demonstrated the oxidation of glycerol under base-free conditions using a gold–platinum catalyst supported on carbon and mordenite. These previous studies have focused on the catalytic oxidation of glycerol at elevated temperatures. Here we show that high glycerol conversion and selectivity to specific C_3_ products (glyceric and tartronic acids) can be achieved at ambient temperatures without employing a base. We further report microstructural characterization of these bimetallic catalysts using aberration-corrected scanning transmission electron microscopy (STEM), high-angle annular dark-field (HAADF) imaging, and energy-dispersive X-ray spectroscopy (XEDS).

Bimetallic catalysts were synthesized by immobilizing colloidal metal particles on an MgO support. X-ray diffraction (XRD) analysis (Supporting Information, Figure S2) revealed that the support material is converted to Mg(OH)_2_ due to hydrolysis during the immobilization process, which is carried out in aqueous solution.

Initial catalyst testing was performed at 60 °C (0.3 mol L^−1^ glycerol solution, glycerol/metal mole fraction 1000, 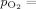
300 kPa, 4 h) with supported Au–Pt (1:3 mole fraction, 1 % by mass total metal loading). A substantially higher activity was observed (Table 1) than has previously been reported for Au–Pt catalysts,^13^ suggesting that the selection of the metal molar ratios and support material for this bimetallic catalyst has a strong influence on activity. The Au–Pt catalyst with 1:3 mol fraction retained significant activity when the temperature was decreased to 40 °C. By extending the reaction time to 24 h and further decreasing the reaction temperature to ambient (23 °C), high conversion was retained with a simultaneous increase in the C_3_ product selectivity (>88 % by mol). In contrast, Au–Pd catalyst prepared with a 1:3 mol fraction and similar metal loading demonstrated significantly lower activity under these conditions, and the selectivity to glyceric acid was lower than that displayed for the Au–Pt catalyst, despite the lower conversion observed. These results suggest that at similar conversion levels, the Au–Pd bimetallic catalysts are significantly less selective to the desired C_3_ products and data at iso-conversion shows this is the case (Table S1).

**Table 1:** The oxidation of glycerol under base free conditions using selected gold bimetallic catalysts at low temperatures

				Selectivity [mol % C]				
Catalyst	*T* [°C]	*t* [h]	Conv. [mol %]	Oxalic acid	Tartronic acid	Glyceric acid	Glycolic acid	Formic acid/CO_2_
AuPd(1:1)/MgO^[a]^	60	4	5.9	0.4	13.3	74.2	4.0	8.1
AuPt(1:1)/MgO^[a]^	60	4	29.2	0.4	12.3	78.4	3.9	5.0
AuPd(1:3)/MgO^[a]^	60	4	14.5	0.1	12.5	74.4	6.4	6.6
AuPt(1:3)/MgO^[a]^	60	4	42.9	0.4	14.9	72.2	3.3	9.2
AuPt(1:3)/MgO^[a]^	40	4	29.4	0.4	9.4	80.4	3.9	5.9
AuPt(1:3)/MgO^[b]^	ambient (23)	24	42.5	0.2	3.8	85.1	4.8	6.1
AuPd(1:3)/MgO^[b]^	ambient (23)	24	29.7	0.7	8.5	66.7	11.5	12.6

[a] Reaction conditions: catalyst metal ratios by mole fraction with 1 % metal loading by mass, water (10 mL), 0.3 mol L^−1^ glycerol, mole fraction of glycerol/metal=1000, CO_2_ was found to be negligible (see Supporting Information). [b] Mole fraction of glycerol/metal=500, *p*(O_2_)=300 kPa, products expressed as mol % C.

Time-on-line data for the reaction at ambient temperature is shown in Figure 1. It is apparent from the reaction profiles that the Pt-containing catalysts are considerably more active than the Pd-containing catalysts at all reaction times. Furthermore the rate exhibits no discernable reduction as the reaction proceeds; suggesting that with extended reaction times greater conversion levels could be achieved. The Au–Pt catalyst also displays significantly higher selectivity to glyceric acid, when compared with Au–Pd catalysts (Table S1). Specifically, for catalysts exhibiting 30 % conversion, Au–Pt gives 85 % selectivity to glyceric acid, while the selectivity of Au–Pd is 67 %. In the reaction with MgO alone less than 0.8 % conversion was observed after 4 h.

**Figure 1:**
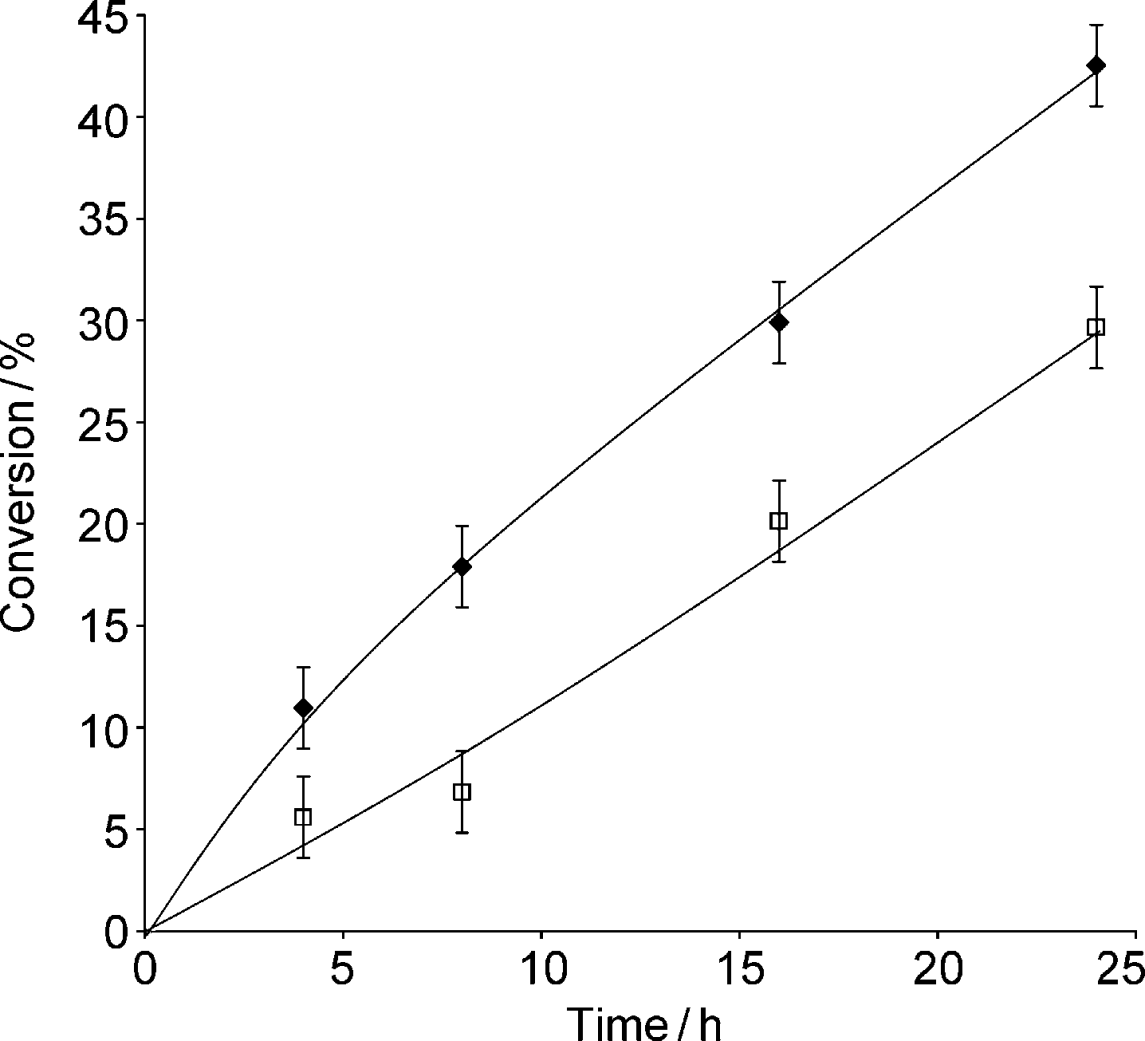
Time-on-line data for selective oxidation of glycerol by Au–M/MgO catalysts, with M=Pt (⧫) or Pd (□). Reaction conditions: 1:3 molar fraction Au:M, total metal loading 1 % by mass, water (10 mL), 0.3 mol L^−1^ glycerol, mole fraction of glycerol/metal=500, *T*=ambient, 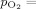
300 kPa. Data points represent the mean value obtained from 10 reaction runs, and the error bars are defined by the range of these measurements.

It is possible that MgO may be acting as a sacrificial base during the reaction and hence we investigated possible leaching of Mg^2+^ during reaction but found it to be negligible (77 ppm Mg^2+^). Indeed, the [Mg^2+^] is 2–3 orders of magnitude lower in amount than the products observed. Furthermore addition of this minimal level of base did not significantly affect the conversion and there was no reaction in the presence of Mg^2+^.

Transmission electron microscopy (TEM) measurements demonstrate that the Au–Pd catalysts have a broader particle-size distribution than the Au–Pt catalysts (Figure S3). The microstructure of the bimetallic catalysts was also investigated using aberration-corrected STEM. The binary phase diagrams of the Au–Pt and Au–Pd systems indicate that, whereas Au–Pd exhibits a complete solid solution at all temperatures below the melting point, Au–Pt exhibits a large miscibility gap with limited solubility between the two face-centered cubic (fcc) end member phases at ambient temperatures.^15^ However, it is well known that bulk phase relations can be substantially altered for nanoscale particles.^15^ In order to determine if the ca. 2 nm particles are homogenous or phase-separated, aberration-corrected STEM-XEDS spectrum imaging was performed. The results for the 1:1 mol fraction AuPt/MgO catalyst (Figure 2) show that there is a good spatial correspondence of the Au and Pt elemental maps formed with the intensities of the L_α_ lines. These results suggest that the individual metal particles do contain both elements. However, this analysis is complicated by the inherently low XEDS signal collected from such small particles, pathological overlaps between the Au-M_α_ and Pt-M_α_ peaks, and the degradation of the underlying Mg(OH_2_) support under the electron beam. Whether the metal particles are homogeneous or phase-separated within each particle (e.g. a core–shell morphology similar to that observed for Au–Pd) could not be resolved due to the extremely small size of the particles and the difficulty in obtaining spatially resolved XEDS data from such small particles at near atomic resolution.

**Figure 2:**
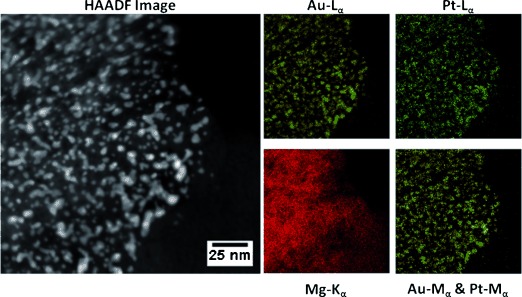
HAADF image of the AuPt(1:1)/MgO catalyst (dried at 120 °C), along with the corresponding Au-L_α_, Pt-L_α_, Mg-K_α_, and (Au-M_α_+Pt-M_α_) elemental maps from the same area.

A representative selection of STEM-HAADF images of the Au–Pd and Au–Pt (both 1:3 mole fraction) particles supported on MgO are presented in Figure 3. The sol-immobilized Au–Pd particles (Figure 3 a,b) were found to have a mixture of characteristic cuboctahedral, decahedral and icosahedral particle morphologies. The latter two particle morphologies, which are multiply twinned crystals that preferentially expose {111}-type surface facets, were dominant. By virtue of the large difference in atomic number (*Z*) between Pd (*Z*=46) and Au (*Z*=79), the Au–Pd particles were determined to be homogeneous. If these particles experienced substantial core–shell segregation, it would have been clearly visible because the HAADF cross-sections of the two elements differ by more than a factor of 2.^16^–^18^ In contrast, STEM-HAADF is insensitive to such segregation in the Au–Pt particles, since Pt and Au have consecutive atomic numbers. The vast majority of Au–Pt particles exhibited a simple cuboctahedral structure, as illustrated in Figure 3 c,d, exposing a mixture of {111} and {200} facets. XEDS point spectrum was acquired by scanning the beam over one particle and therefore the averaged composition information of individual particle can be obtained. As shown in Figure S4 and S5, particles from both sides of the size distribution are all alloyed. However in the Au–Pd system there is a size-dependant composition variation, which we have reported previously:^19^ the relative ratio of Au-Mα line and Pd-Lα line showed that Pd tends to agglomerate on bigger particles. Interestingly, Au–Pt system seemed to not have such an obvious segregation trend, with the two metals distributed more evenly.

**Figure 3:**
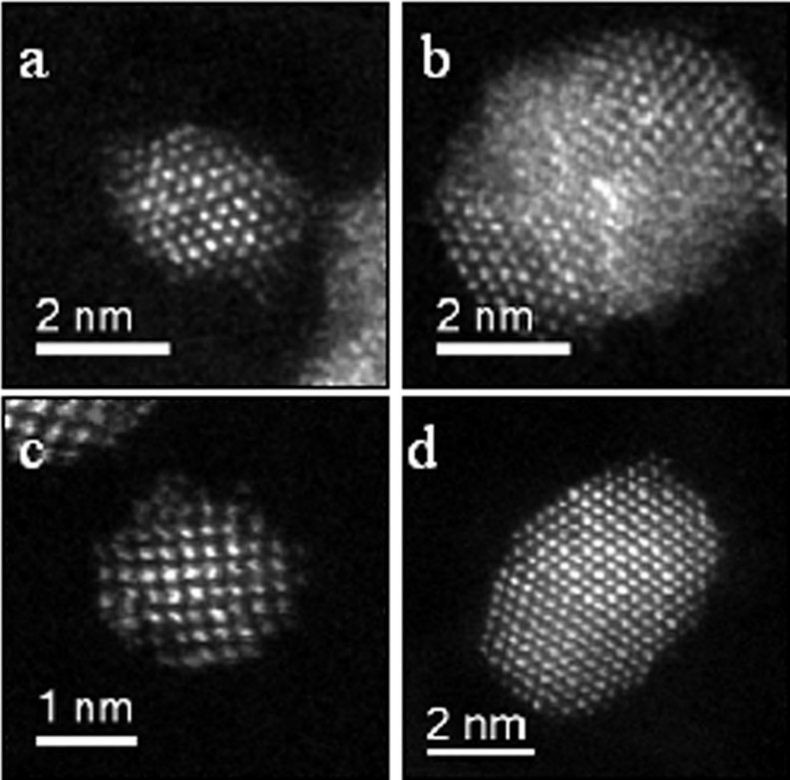
High-magnification STEM-HAADF image and corresponding particle-size distribution of a,b) AuPd(1:3)/MgO and c,d) AuPt(1:3)/MgO.

Finally, we explored the reusability of the 1:3 molar ratio AuPt/Mg(OH)_2_ catalyst both for reactions at ambient temperature and at 60 °C (Figure S6 and Table S2). After recovering the used catalyst by filtration, the activity for glycerol oxidation was found to be unchanged within experimental error and reusable under these conditions. Furthermore, we have demonstrated the more general use of AuPt/MgO as a catalyst for alcohol oxidation under base-free conditions by reacting ethylene glycol, 1,2-propanediol and 1,4-butanediol and the catalyst is shown to be effective (Tables S3–S5). In addition we have successfully shown that the catalyst remains highly effective at a higher concentration of glycerol of 1.2 m (Table S6).

In summary, Au–Pt and Au–Pd nanoparticles supported on Mg(OH)_2_ are highly active for the selective oxidation of glycerol under base-free conditions at ambient temperature. This reactivity at ambient temperature under base-free conditions emphasises the activity of these AuPt/MgO catalysts. Within an appropriately chosen compositional range, catalysts with a narrow particle size distribution can be produced. When alloyed with Au, Pt leads to both enhanced glycerol conversion and selectivity towards C_3_ products relative to Pd. These Au–Pt catalysts may permit bench-top reaction procedures for alcohol oxidation to be more commonly practiced in synthetic methodology.

## Supplementary material

Detailed facts of importance to specialist readers are published as ”Supporting Information”. Such documents are peer-reviewed, but not copy-edited or typeset. They are made available as submitted by the authors.
