# Supplementary material for: Selective Oxidation of Glycerol by Highly Active Bimetallic Catalysts at Ambient Temperature under Base-Free Conditions
Source: Angew Chem Int Ed Engl. 2011 Aug 24;50(43):10136–9. doi: 10.1002/anie.201101772 (PMC3644171; doi:10.1002/anie.201101772)
Supplement: Supplementary file 1 [file anie0050-10136-SD1.pdf]

Angewandte Chemie
Eine Zeitschrift der Gesellschaft Deutscher Chemiker

Supporting Information

© Wiley-VCH 2011

69451 Weinheim, Germany

Selective Oxidation of Glycerol by Highly Active Bimetallic Catalysts at Ambient Temperature under Base-Free Conditions**

*Gemma L. Brett, Qian He, Ceri Hammond, Peter J. Miedziak, Nikolaos Dimitratos, Meenakshisundaram Sankar, Andrew A. Herzing, Marco Conte, Jose Antonio Lopez-Sanchez, Christopher J. Kiely, David W. Knight, Stuart H. Taylor, and Graham J. Hutchings**

anie_201101772_sm_miscellaneous_information.pdf

Experimental Methods

1. Catalyst preparation

Au-Pd and Au-Pt catalysts supported on carbon and magnesium oxide were prepared using a sol-immobilisation method. Aqueous solutions of $PdCl_2$ (Johnson Matthey) † or H_2PtCl_6 (Johnson Matthey) and $HAuCl_4.3H_2O$ (Johnson Matthey) of the desired concentration were prepared. To this solution polyvinyl alcohol (PVA) (weight-average molecular mass = (9,000 to 10,000 g/mol)), 80 % hydrolysed, Aldrich) was added such that the PVA to Au ratio was 1.2 by mass. Subsequently, a freshly prepared 0.1 M solution of $NaBH_4$ (> 96 % purity, $NaBH_4$/Metal mole fraction = 5, Aldrich) was added to form a dark-brown sol. After 30 min of sol generation, the colloid was immobilised by adding activated carbon (Aldrich, G-60) (acidified to pH 1 by sulfuric acid) or magnesium oxide under vigorous stirring. The amount of support material required was calculated to give a final metal loading of 1 % by mass. After 2 h, the slurry was filtered, the catalyst washed thoroughly with distilled water and dried at 120 °C overnight. Further catalysts were prepared using a 3:1 mole fraction and 1:3 mole fraction of Au:Pd or Au:Pt.

2. Glycerol oxidation

Catalytic reactions were carried out using a 50 mL glass reactor. The glycerol solution (0.3 mol/L) was admitted into the reactor and the desired amount of catalyst (glycerol/metal mole fraction = (500 to 1000)) was suspended in the solution. The glass reactor was purged with oxygen three times and adjusted to the desired pressure of 300 kPa. This pressure was maintained at a constant level throughout the experiment; hence as the oxygen was consumed in the reaction it was continuously replenished. The reaction mixture was heated to the desired temperature (23 °C to 60 °C) and stirred for the requisite amount of time (between 4 h and to 24 h). The reactor vessel was then cooled to room temperature and the reaction

mixture analysed. This analysis was carried out using high-pressure liquid chromatography (HPLC) equipped with ultraviolet and refractive index detectors. Reactants and products were separated using a Metacarb 67H column eluted with 0.01 mol/L aqueous H_3PO_4 with a flow rate of 0.3 mL/min. Samples of the reaction mixture (0.5 mL) were diluted (to 5 mL) using the eluent. Products were identified by comparison with authentic samples, and an analysis of the experimental error over multiple reactions runs has shown that the values presented in Table 1 and Figure 1 are reported within $\pm$ 2 %. For the quantification of the reactants consumed and products generated, an external calibration method was used.

CO_2 analysis

The amount of CO_2 produced during the reaction was determined as follows. The oxidation of glycerol was carried out in a stainless-steel autoclave (Parr autoclaves) with a total volume of 50 mL. The vessel was charged with an aqueous solution of glycerol (10 mL, 0.3 M glycerol) which was thoroughly degassed with nitrogen prior to use in order to expel residual CO_2 from the solution and the desired amount of catalyst was added. After sealing, the reactor was charged with O_2 to a fixed pressure (42 psi) after a series of purges (5 times with O_2 at 140 psi) removed the contaminant gasses. The autoclave was heated to the reaction temperature (60 °C), and vigorously stirred at 1500rpm once the desired temperature was obtained. The reaction was carried out for 2 h, after which the vessel was cooled using an ice/water bath to a temperature of 12 °C. The resultant solution was filtered and analysed by a combination of HPLC and GC-FID.

Analytical

Gas phase. The gaseous phase products were analysed by an FID on a Varian 450-GC fitted with a CP-Sil 5CB capillary column (50m length, 0.32mm ID). The GC was fitted with a methaniser unit, and CO_2 was quantified against a calibration curve constructed from

commercial standards (BOC gasses). The CO2 analysis showed negligible formation of CO_2 (0.44 mol%).

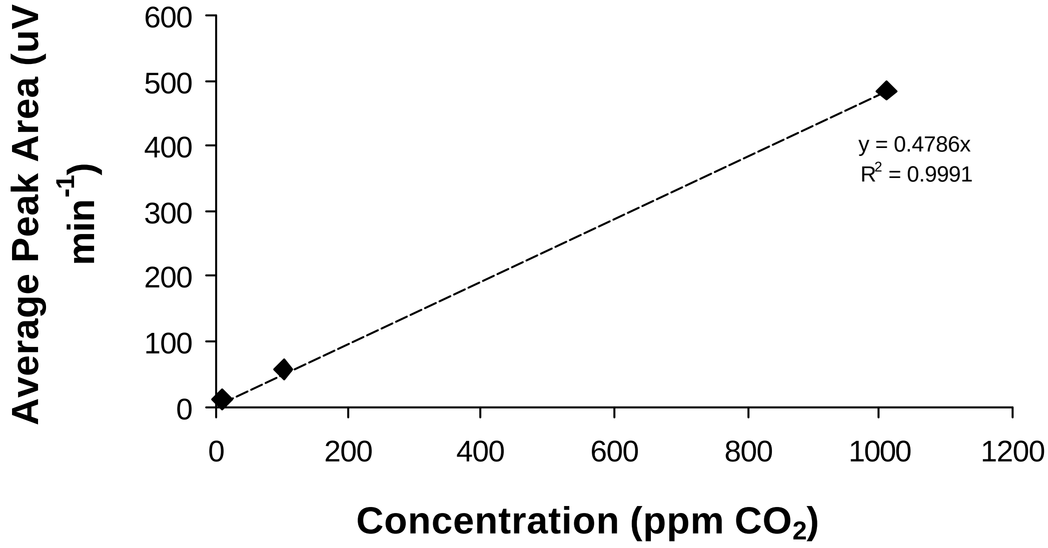

Figure S1: Calibration curve for CO_2 quantification.

2.1 Catalyst reuse

Catalytic reactions were carried out as described previously but with a glycerol/metal ratio of 250 mol/mol. After the reaction the catalyst was filtered (gravity), washed with distilled water and dried overnight at ambient temperature. This catalyst was then tested as described above.

3. Catalyst characterisation

3.1 X-ray powder diffraction

X-ray diffraction (XRD) was performed using the Cu K_a radiation on a PANalytical X'PertPRO X-ray diffractometer operated at 40 kV and 30 mA. X-ray patterns were baseline corrected and subjected to Gaussian smoothing to improve the signal-to-noise ratio.

3.2 Transmission Electron Microscopy

Samples for scanning transmission electron microscopy (STEM) analysis were prepared by dipping a carbon-coated copper TEM grid directly into the finely ground dry catalyst powder and then shaking off any loosely bound residue. Scanning transmission electron microscopy (STEM) high angle annular dark field (HAADF) imaging was used to image the individual nanoparticles at atomic resolution with an aberration corrected JEOL 2200FS (S)TEM operating at 200 kV, in addition with the capability of taking XEDS spectra from individual particles larger than 1-2 nm. STEM-XEDS spectrum imaging was performed in an aberration-corrected FEI Titan 80-300 (S)TEM equipped with a 4pi Revolution EDX system. Spectrum images were collected by integrating the intensities from 3200 frames using a per-pixel dwell-time of 32 μs, resulting in a total per-pixel acquisition time of ˜ 100 ms. Spatial drift was corrected between each frame by on-the-fly cross correlation analysis of HAADF images, which were acquired simultaneously with the XEDS spectrum image. Elemental maps were extracted using NIST Lispix version 157P.

† - Certain commercial equipment, instruments, or materials are identified in this document. Such identification does not imply recommendation or endorsement by the National Institute of Standards and Technology, nor does it imply that products identified are necessarily best available for the purpose.

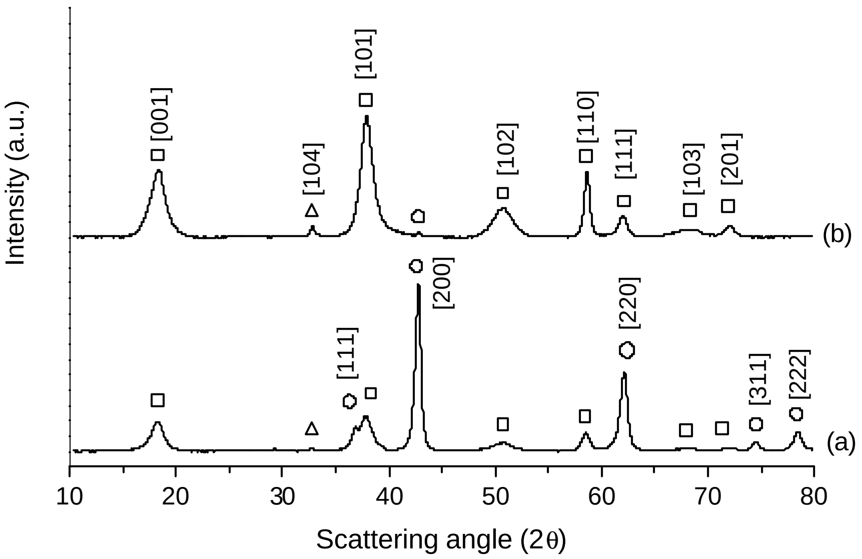

Figure S2: XRD characterization of (a) the bare MgO support, and (b) a Au-Pd(1:1)/MgO catalyst dried at 120 °C. In square brackets are reported the Miller indices of the reflections of (?) MgO periclase, (?) $Mg(OH)_2$ brucite, and (?) $MgCO_3$ magnesite.

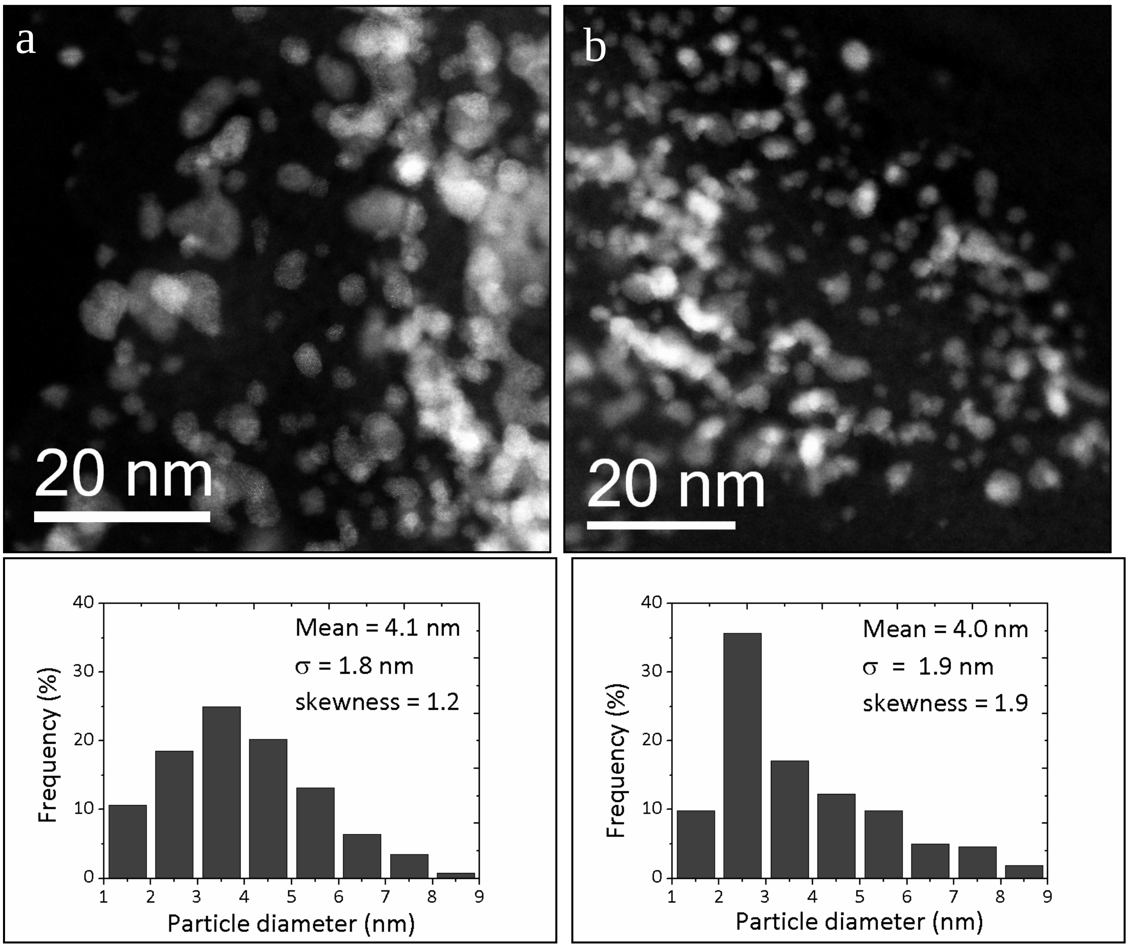

Figure S3: Low mag STEM-HAADF image and corresponding particle size distribution of the (a) AuPd(1:3)/MgO and (b) AuPt(1:3)/MgO.

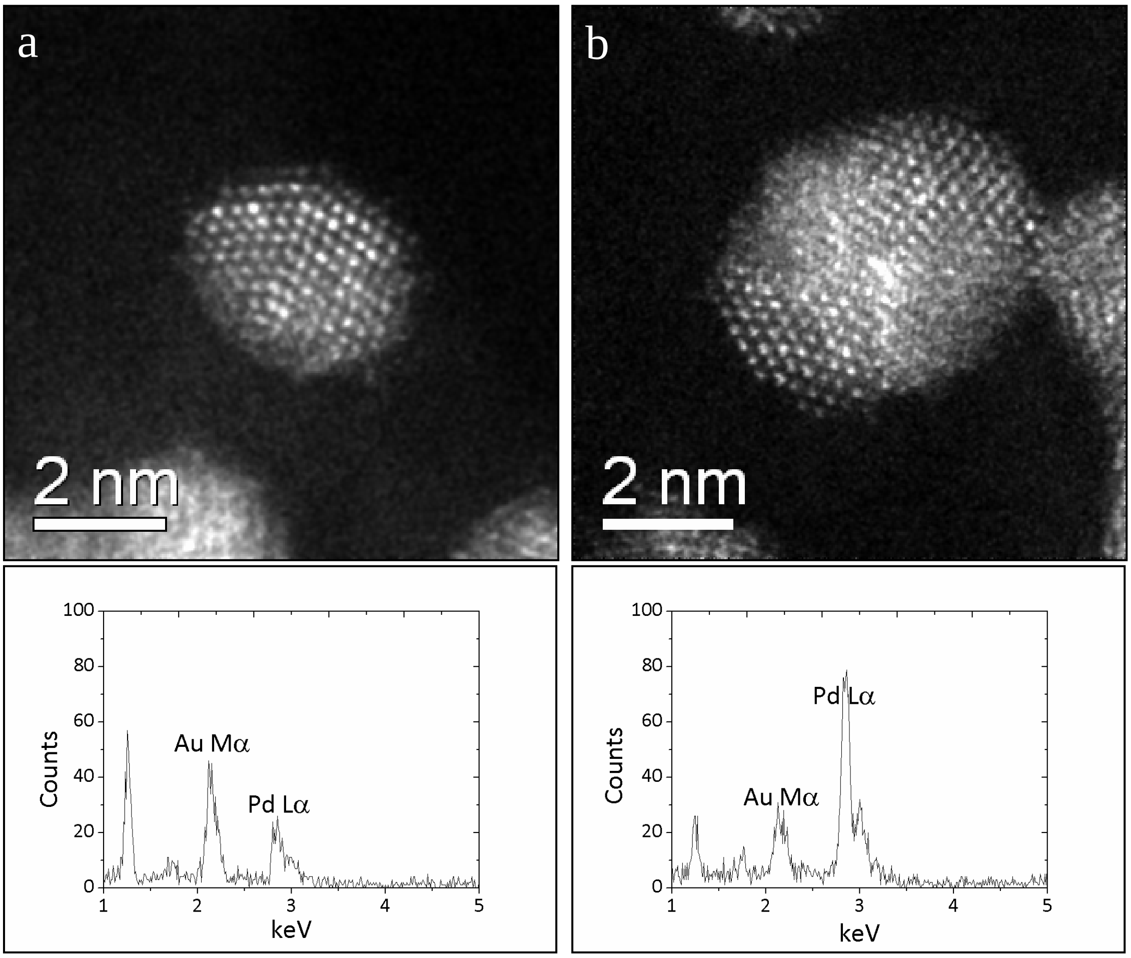

Figure S4: High mag STEM-HAADF images and corresponding X-ray energy dispersive spectra of the individual nanoparticles in AuPd(1:3)/MgO. (a) 2 nm particle; (b) 6 nm particle.

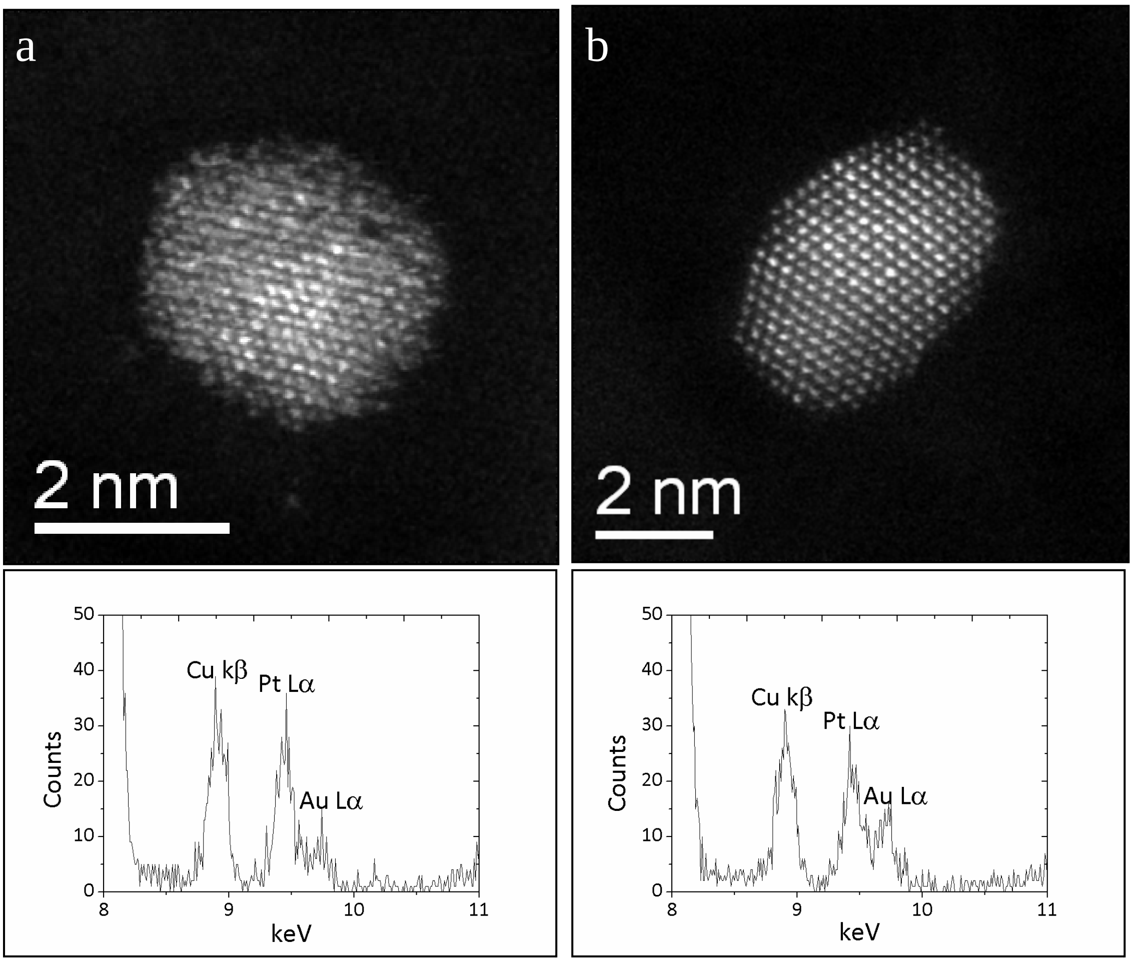

Figure S5: High magnification STEM-HAADF images and corresponding X-ray energy dispersive spectrum of the individual nanoparticles in AuPt(1:3)/MgO. (a) 2 nm particle; (b) 6 nm particle.

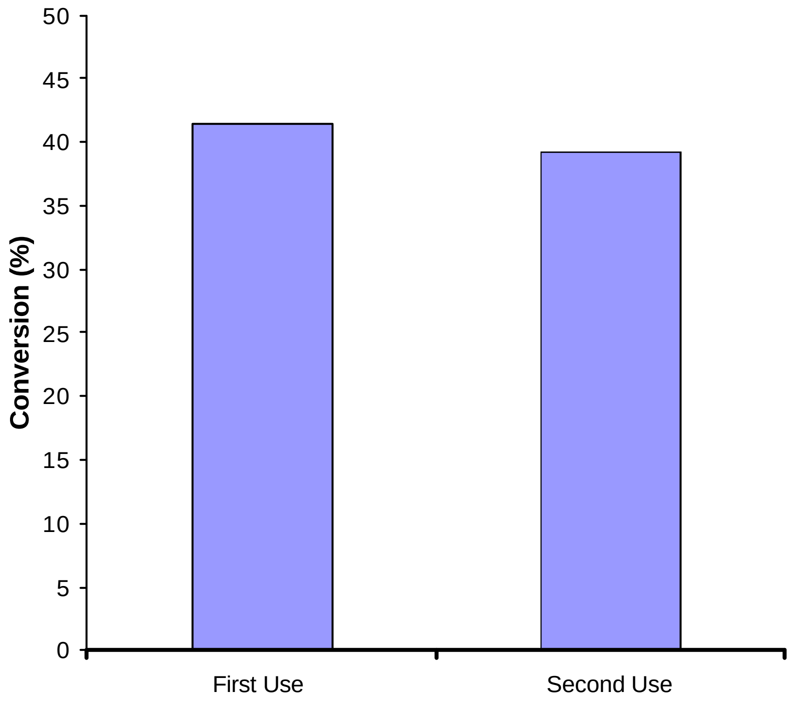

Figure S6: Re-use data of the $Mg(OH)_2$-supported catalyst containing an overall metal loading of 1 % by mass and a 1:3 mole fraction of Au to Pt. Reaction conditions: 10 mL of 0.3 mol/L glycerol, mass fraction of glycerol/metal = 500, pO_2 = 300 kPa, time of reaction = 24 h at ambient temperature (23 °C).

Table S1: Iso-conversion data for the oxidation of glycerol under base free conditions using selected gold bimetallic catalysts at low temperatures.[a]

Catalyst	T [°C]	Conv. [mol%]	Selectivity [mol C%]				
			Oxalic acid	Tartronic acid	Glyceric acid	Glycolic Acid	Formic Acid
1:3M Au-Pd/MgO	Ambient (23)	30	0.7	8.5	66.7	11.5	12.6
1:3M Au-Pt/MgO	Ambient (23)	30	0.2	3.7	84.9	5.0	6.2

[a] Reaction conditions: Catalyst 1:3 mole fraction Au:M/MgO (M = Pt or Pd) with 1 % metal loading by mass, water (10 ml), 0.3 mol/L glycerol, mole fraction of glycerol/metal = 500, pO_2 = 300 kPa.

Table S2: The oxidation of glycerol under base free conditions using AuPt/MgO: Resusability tests.[a]

Catalyst	T [°C]	Time [h]	Conv. [mol%]	Selectivity [mol C%]				
				Oxalic acid	Tartronic acid	Glyceric acid	Glycolic Acid	Formic Acid/CO_2
1:3M Au-Pt/MgO Fresh	60	0.5	20.5	0.3	2.6	87.7	5.8	3.6
1:3M Au-Pt/MgO used once	60	0.5	18.7	0.8	14.7	75.2	2.0	7.3
1:3M Au-Pt/MgO used three times	60	0.5	19.1	1.2	23.8	66.4	2.1	6.5

[a] Reaction conditions: Catalyst 1:3 mole fraction Au:Pt/MgO with 1 % metal loading by mass, water (10 ml), 0.3 mol/L glycerol, mole fraction of glycerol/metal = 500, pO_2 = 300 kPa.

Table S3: The oxidation of ethylene glycol under base free conditions using AuPt/MgO[a]

Catalyst	T [°C]	Time [h]	Conv. [mol%]	Selectivity [mol C%]	
				Oxalic acid	Glycolic acid
1:3M Au-Pt/MgO Fresh	60	4	40.6	24.5	75.5

[a] Reaction conditions: Catalyst 1:3 mole fraction Au:Pt/MgO with 1 % metal loading by mass, water (10 ml), 0.3 mol/L ethylene glycol, mole fraction of glycerol/metal = 500, pO_2 = 300 kPa.

Table S4: The oxidation of 1,2-propanediol under base free conditions using AuPt/MgO[a]

Catalyst	T [°C]	Time [h]	Conv. [mol%]	Selectivity [mol C%]			
				Hydroxyacetone	Lactic acid	Acetic acid	Others[b]
1:3M Au-Pt/MgO Fresh	60	4	40.6	44.7	14.8	8.9	31.6

[a] Reaction conditions: Catalyst 1:3 mole fraction Au:Pt/MgO with 1 % metal loading by mass, water (10 ml), 0.3 mol/L 1,2-propanediol, mole fraction of substrate/metal = 500, pO_2 = 300 kPa. [b] Others including CO_2 and formic acid.

Table S5: The oxidation of 1,4-butanediol under base free conditions using AuPt/MgO[a]

Catalyst	T [°C]	Time [h]	Conv. [mol%]	Selectivity [mol C%]
				?-butyrolactone
1:3M Au-Pt/MgO Fresh	60	4	13.3	100

[a] Reaction conditions: Catalyst 1:3 mole fraction Au:Pt/MgO with 1 % metal loading by mass, water (10 ml), 0.3 mol/L 1,4-butanediol, mole fraction of substrate/metal = 500, pO_2 = 300 kPa.

Table S6: The oxidation of glycerol under base free conditions using AuPt/MgO[a]

T [°C]	Time [h]	Conv. [mol%]	Selectivity [mol C%]				
			Oxalic acid	Tartronic acid	Glyceric acid	Glycolic Acid	FormicAcid/CO_2
60	0.5	4.1	0.1	3.7	72.1	12.5	11.6
60	2	8.5	0.4	8.6	71.8	10.0	9.2
60	24	30.4	0.7	10.8	64.2	13.0	11.3

[a] Reaction conditions: Catalyst 1:3 mole fraction Au:Pt/MgO with 1 % metal loading by mass, water (10 ml), 1.2 mol/L glycerol, mole fraction of glycerol/metal = 2000, pO_2 = 300 kPa.
